# Baccalaureate nursing Students’ perspectives on learning about caring in China: a qualitative descriptive study

**DOI:** 10.1186/1472-6920-14-42

**Published:** 2014-03-04

**Authors:** Fang Ma, Jiping Li, Hongmin Liang, Yangjuan Bai, Jianhua Song

**Affiliations:** 1 Department of Nursing, West China Hospital, Sichuan University, Chengdu, China; 2 Department of Nursing, the First Affiliated Hospital of Kunming Medical University, Kunming, China; 3 Cardiology Department, the First Affiliated Hospital of Kunming Medical University, Kunming, China

**Keywords:** Caring, Baccalaureate nursing students, Focus group, Nursing, Qualitative study

## Abstract

**Background:**

The need to provide humanistic care in the contemporary healthcare system is more imperative now and the importance of cultivating caring in nursing education is urgent. Caring as the primary work of nursing has been discussed extensively, such as the meaning of caring, and teaching and learning strategies to improve nursing students’ caring ability. Yet attempts to understand students’ perspectives on learning about caring and to know their learning needs are seldom presented. The aim of this qualitative descriptive study was to explore the baccalaureate nursing students’ perspectives on learning about caring in China.

**Methods:**

A qualitative descriptive study using focus group interviews were undertaken in two colleges in Yunnan Province, China from February 2010 to April 2010. Purposeful sampling of 20 baccalaureate nursing students were recruited. Content analysis of the transcribed data was adopted to identify the themes.

**Results:**

Four categories with some sub-categories related to students’ perspectives on learning about caring were identified from the data: 1) Learning caring by role model; 2) conducive learning environment as the incentive to the learning about caring; 3) lack of directive substantive way of learning as the hindrance to the learning about caring; 4) lack of cultural competency as the barrier to the learning about caring.

**Conclusions:**

Both caring and uncaring experiences can promote the learning about caring in a way of reflective practice. The formal, informal and hidden curricula play an important role in the learning about caring. Cultural awareness, sensitivity and humility are important in the process of learning to care in a multicultural area.

## Background

In this time of rapid knowledge and technological advances, healthcare professionals must be educated to be not only technologically proficient but also to genuinely care for patients. Caring is a core value in nursing practice and thus the capacity to care is a desired attribute in nursing students, besides appropriate academic qualifications nursing students will have appropriate caring behaviors [1]. Nurturing a caring attitude in nursing education is important as this is the first place for students to learn about the most significant values and essence of their profession [2]. A critical task of nurse educators is to promote nursing students’ learning about caring, hence it is imperative to explore students’ perspectives and thoughts during the process of learning about caring.

As evidenced by practice and research, caring has long been recognized as central to nursing. Latham [3] reported that sensitive and supportive nurse caring contributes to patients’ overall coping effectiveness. Duffy [4] found that nurse caring has association with patient satisfaction. Consequences of caring for the one cared for (most often patient or student) were multiple indicators of enhanced well-being, and practicing in a caring manner leads to the nurse’s well-being, both personally and professionally [5]. As a result of caring, patients experience improvement in physical and mental well-being. Due to the reciprocal nature of caring, nurses report an improved sense of mental well-being [6]. Watson [7] concluded that for patients who experienced caring, outcomes included: emotional and spiritual well-being; enhanced healing and enhanced relationship with others. She also mentioned that for nurses practicing caring, the outcomes were: a sense of personal and professional satisfaction and fulfillment; a love of nursing and the ability to live out their own philosophy [7]. The findings remind the nursing profession of the importance of learning caring because it is critical to the outcomes for patients and nurses.

Due to the pivotal role of caring in nursing profession, the meaning, the essential components and the influences of caring have long been studied. Leininger [8] defines care as “cognitive learned, culturally specific modes of helping others to receive personalized services to improve, maintain a healthy state for life or death” [8].

Nurse caring was reported as an “interactive process that occurs during moments of shared vulnerability between nurse and patient” [9,10]. Swanson [11] defined caring as “A nurturing way of relating to a valued other toward whom one has a personal sense of commitment and responsibility”. Caring has been described as human trait, moral imperative, interpersonal relationship, therapeutic intervention and an affect [12]. In a middle range theory of caring, it consists of five categories: knowing, being with, doing for, enabling, and maintaining belief [11]. Watson [7] concluded that caring consists of 10 Carative factors/Caritas process that facilitate healing, honor wholeness, and contribute to the evolution of humanity. In a Meta-synthesis of caring, the attributes of caring process include expert nursing, interpersonal sensitivity and intimate relationships. The need for and openness to caring of care recipient, professional maturity and moral foundations of nurses, and conducive work environment are reported to be antecedents of caring [6]. Some researches have demonstrated that patients-related, nurse-related and organization-related conditions affect caring [5]. Cultural variations in caring relationship are of particular note. According to Leninger’s cultural care diversity theory, culture and care were embedded in each other and caring needs to be teased and understood in a cultural context [13]. In a cross-cultural study of the concept of caring through behaviors, important differences were observed between patients’ and nurses’ perceptions of nurse caring behaviours. Their views on the four factors of caring: assurance of human presence, knowledge and skills, respectful deference to others and positive connectedness were widely diverse in six different EU countries [14]. The many researches support that caring is a complex phenomenon and the concept of caring has not been clearly conceptualized, which make the learning of caring more difficult. Therefore, it is important to identify students’ perspectives on learning about caring so that educators can know their learning needs, thus ways can be found out to facilitate students’ learning about caring.

In Yunnan Province, south west China, there are 26 nationalities living together. Each brings a different worldview, unique custom and lifestyle, and different religion. Confucianism, Buddhism, Taoism, Christianism, Catholicism, Muslim and some indigenous religions exist in this region. Influenced by the cultural diversity in this particular region of China, people here may have different definitions of what constitutes health and illness, how illness should be managed, how care should be expressed and the framework of caring [15,16]. In China, caring is only sparsely investigated in limited geographic locations from a nurse’s nursing perspective [17], there is a scarcity of research concerning students’ perspectives on learning about caring. Therefore, when nursing students’ perspectives on learning about caring in such a multicultural area are revealed, nursing educators, practitioners and administrators may be further aware of what students really need and in which way to help them learn about caring in practice. In this qualitative study, the purpose was to explore baccalaureate nursing students’ perspectives on learning about caring, which will aid the understanding of learning about caring from the students’ point of view and gain insight into the way to improve students’ caring ability.

## Methods

### Study design

A qualitative descriptive study using 4 focus groups interviews was adopted in this study. The aim of this study was to explore the perspectives of baccalaureate nursing students on learning about caring in two colleges of Yunnan Province.

### Setting and participants

In Yunnan Province, there were 2 medical colleges which have established 4-year, full-time Bachelor of Nursing programme. One college is located in the center of the province and another one is in the south of the province. As for the two sites, the one located in the center is the capital city of Yunnan Province, the other one located in the south once served as the seat of government and a major military barracks for Yunnan Province in ancient times. And it is a gateway to the Silk Road in southwest China, which is a historically important international trade route between China and the Mediterranean. Besides, many ethnic minority groups live in the southern site. There are geographical, cultural and historical differences between the two sites, which may result in different learning atmosphere and provide the chance for students to have a high degree of contact with patients from different cultural backgrounds. We hope that we can obtain as heterogeneous sample as possible by choosing students with various experiences in life and study from the two different sites.

A purposeful sample of 20 full-time baccalaureate nursing students from the two colleges were chosen to obtain cases deemed information-rich for the purpose of study [18]. Participants who have experienced the phenomena of interest is essential to obtaining detailed meaningful description. Only students who have been in clinical settings and have had the opportunity to observe or experience caring in the healthcare setting may provide rich and detailed information. Students who had practical experiences in clinical settings and were recommended by their teachers as talkative, outspoken, thoughtful were selected in this study. Participation was voluntary and a written invitation was sent to the selected students which explained the purpose and procedure of the study. 20 students responded to the invitation and participated the 4 focus groups with two in each college. The sample consisted of three male students and seventeen female students. There were one Hui minority, one Yi minority and eighteen Han people. They were in year 3 and 4, referred to as clinical stage, which meant they had been in clinical settings and experienced the phenomena of learning about caring in a profound way. Ages ranged from 18 to 25 years, with the average age being 22 years. In the study population, the majority belonged to Han ethnic, and the rest ethnic minorities were composed of Hui, Yi, Bai and Dai ethnic minority. Among the selected students, only two ethnic minority students responded to the invitation. The low representation of ethnic minority students in the sample was consistent with the composition of the population.

### Data collection

Four focus groups interviews with 5 participants in each group were conducted in two colleges respectively, with two focus groups in each college. As one of the widely used qualitative interview methods, focus group interviews can contribute to a body of knowledge that is conceptual and theoretical [19], and they can be used to elicit the student perspective in medical education [20]. Besides, focus groups are a useful tool to expand existing knowledge particularly within multicultural populations [21]. Two researchers with experience in qualitative research methods conducted all the four focus groups interviews. Both researchers had no supervisory relationship to the students and they were assured that their responses would not affect their grades. In each interview, one researcher facilitated the discussion and the second mainly took notes of participants’ tones, facial expressions, body language, and their interactions in the groups in order to provide more contextual information for subsequent analysis. The following topics guided the focus groups: (1) How do you learn about caring? (2) What factors facilitate and hinder your learning about caring. The focus group interviews lasted approximately one hour for each, and were audio-taped, from February 2010 to April 2010. Participants were asked to verify the accuracy of the information discussed during the interview before the end of the interview. Using the focus group method allowed the participants to relate and react to each other’s experiences, even induced some debate which helped them clarify their thoughts and feelings. The facilitator encouraged the participants to talk freely and valued different opinions, which contributed to developing an open and non-threatening environment for discussion, thereby creating a synergy of group talk that individual interviews could not offer.

### Ethical considerations

The study was approved by the ethics committees of the colleges. Participants were assured that their names would not be used and confidentiality would be maintained by the researchers. Participants were reminded that all information shared in the focus groups was confidential. Before data collection informed consent was obtained from each participant. Participants were told to have the right to quit during the group discussion because the participation was to be voluntary.

### Data analysis

The data were analyzed using conventional content analysis, which is generally used to provide a description of a phenomenon about which little is known in existing theory or literature [22]. All tape-recorded interviews were transcribed verbatim immediately after the group discussion. The process of analysis included listening to the audiotape, thoughtful reading and rereading of the transcribed texts, listening again to the tape-recorded discussion to spot some meaningful hints. All data were read repeatedly to achieve immersion and obtain a sense of the whole [23]. Then, data were read using line-by-line analysis to identify codes appearing to capture key thoughts or concepts of the interview discussion. The various codes were compared based on differences and similarities and sorted into sub-categories and categories, which were organized by the research areas defined in the focus group discussion guide. The point of view or voice that prevailed in the interviews were considered [24], and the techniques of data analysis and data re-presentation pointed out by Sandelowski [18] were used in the data analysis. To ensure qualitative rigour and trustworthiness of the data, two researchers analyzed the data separately. The interviewer (FM) analyzed the focus group interview text and another researcher (JS) with knowledge in qualitative analysis who did not attend the focus groups analyzed the data, communicated with the primary researchers about the analysis to assure that an inductive process occurred and that was consistent with the views of the participants of the study. Agreement was reached through discussion where differences in analysis appeared.

## Results

Data analysis of this study resulted in the identification of four categories with some sub-categories answering the guided research questions: 1) Learning caring by role model; 2) conducive learning environment as the incentive to the learning about caring; 3) lack of directive substantive way of learning as the hindrance to the learning about caring; 4) lack of cultural competency as the barrier to the learning about caring.

### Category1: learning caring by role model

The students discussed how they learned to care and all participants were adamant that they learned about caring by role model. The prevailing sub-category was that the positive role models such as caring incidents facilitated the willing of caring and provided the knowledge, skills, tips for the learning about caring. Yet, as for the opposite situation---negative role models such as uncaring experiences, it was mentioned in one focus group (Group 3), which involved both agreement and disagreement about their constructive function in the learning about caring. In the end, the participants in group 3 came to a conclusion that uncaring experiences also acted as a way to learn to care, thus this category comprised two sub-categories: 1) positive role model--- an ideal way of learning about caring; 2) negative role model--- another way of learning about caring.

#### Sub-category 1: positive role model--- an ideal way of learning about caring caring

Incidents acted as positive role models were depicted by the participants as observing or experiencing caring encounters such as caring relationships and behaviors around them. In all the 4 focus groups, the participants pointed out that they learned to care by caring experiences. They stated that caring experiences acted as a vivid class of the demonstration of caring which could teach how to care for others in the real context like positive role models, additionally they were a source of motivation for the learning about caring. As quoted by participant 2 in focus group 4:

“When I practiced in the cardiology department, my preceptor was really a warm-hearted, competent and caring person. By observing how she worked, I considered her as a positive role model and I felt the caring atmosphere. By imitating what she did in the practice, I learn how to provide nursing care in a caring way in the ward which can’t be learned in books”. (Group 4)

Another participant (participant 3 in focus group 3) recounted her experience of caring experiences as the motivation for the learning about caring:

“During my clinical rotation in the respiratory department, a newly admitted patient spoke out my name and smiled at me, but I did not recognize him at first. Then he said I cared for him in the gastrointestinal department three months ago, and he expressed his gratitude for me then asked if I could continue to care for him. It was rewarding and I received the sense of caring from my clients. This experience reminded me of the good side of life, …caring is reciprocal, which makes me full of courage to learn to care for others”. (Group 3)

#### Sub-category 2: negative role model--- another way of learning about caring

Contrary to caring incidents, uncaring incidents were described by the participants as uncaring behaviors such as being rude to patients and to students alike, not displaying empathy, concern, commitment, and horizontal violence in the medical team which were like negative role models. In group 3, when asked how they learned to care, besides leaning caring by caring experiences, one participant (participant 1) pointed out she also learned caring by uncaring experiences. A different voice of the role of uncaring experiences was heard and discussed. Firstly, some participants stated that uncaring experiences showed the negative role models for the learning about caring and might hinder their learning of caring. Then participant 1 put forward a different point of view by her own experience of learning caring by uncaring experience.

“Once I was sick and coughed frequently in my clinical rotation, seeing this, my preceptor immediately wore two masks and avoided to talk to me. She seemed to cold-shoulder me and a sense of rejection and isolation surrounded me. I was sad … After the gloomy moment, I told myself that in the future I would never treat the patients like I was treated by the teacher. Since they suffer pain, I will give more caring to them. It is uncaring experiences that highlight the importance of caring and improve our motivation and willingness to care and to learn about caring”. (Group 3)

There occurred a debate as for the role of uncaring incidents. Some participants described this kind of learning by uncaring incidents as unusual and impossible (as evidenced by the high pitch of their tones). But when another participant (participant 3) cited her experience of uncaring incident, most of the participants’ views began to change.

“Once I was in the Gynecological out-patient department, some female patients were shy and answered the medical staffs’ questions in a low voice, whereas the staff would ask them in a loud and impatient tone which made patients uneasy and embarrassed. Observing this uncaring incidents, I talked with my mentor about this negative experience, she encouraged me to analyze this incident deep, and we had a critical dialogue concerning this negative experience. Then I think if it was me that received the patients, I would had asked in a low and soft voice, which could make them in a more comfortable condition. Such uncaring experience make me understand the true meaning of ‘sensitivity to human suffering’, and can facilitate the learning about caring. Think again, maybe you have the same experience” (Group 3)

Hearing this, there was a moment of silence and some participants were nodding. Then they proceeded to deep discuss this topic, and agreed that it was often uncaring experiences instead of caring experiences that impressed them most and facilitated their understanding and articulation of caring. It was also uncaring experiences that invoked their thinking and generated the most learning, but they also pointed that too many uncaring incidents might make them in a state of apathy, even cause moral distress, which would hinder their learning about caring. At this moment, dialogue with competent mentors/supervisors would help them analyze and reflect upon these negative experiences, which might provide support and guidance for them.

#### Category2: conducive learning environment as the incentive to the learning about caring

When talking about the facilitators of the leaning about caring, participants in the four focus groups mentioned the conducive learning environment contributed to the learning about caring. Conducive learning environment were described in the focus groups as caring teachers and preceptors (role modeling), caring learning environment and the microsystem that valued caring over simple curing and task completion. It was mentioned by all the participants that the caring teachers and preceptors as role modeling will guide and facilitate their learning about caring. As for the role of caring learning environment, participant 3 in group 2 stated:

“In a caring learning environment, we can have support from the team and reduce stressful learning conditions, which benefits the process of learning to care”. (group 2). In the focus group interviews, some participants pointed out that the microsystem was key to the learning process. In focus group 2, the students compared the different clinical practice placements, and concluded that the placement addressed caring was an optimal place for the leaning about caring, as participant 2 in focus group 2 stated:

“If the department values caring as important, we will have more resources for learning to care”. (group 2)

#### Category3: lack of directive substantive way of learning as the hindrance to the learning about caring

Most of the participants ( in group 1, 2, 3) indicated that caring should and could be taught and learned. It should not be taken for granted that nursing students could care for people just because they would enter into the nursing profession. They considered that in China today, the importance of humanistic caring was emphasized in nursing, but caring theory, knowledge, skills, attitudes, the art of caring were lacking in nursing education and there were no caring related courses or program during their learning process, and solutions should be found out. As participant 2 in focus group 1 stated:

“Teachers and supervisors often tell us the importance of caring in our future career life, but little is mentioned about how to care and we are seldom taught about these knowledge. The delivery of care in clinical practice includes caring related theory, knowledge, skills, attitudes, the art of caring…, all these should and can be taught and learned in school as well as in practice. The profession cannot empower us with the ability to provide care for patients from the beginning, we must be trained first”. (group 1)

#### Category 4: lack of cultural competence as the barrier to the learning about caring

In all focus groups, both Han and the ethnical minority participants talked the cultural issues as their confusing concern during the practice and learning process of caring. They mentioned the cultural conflict of health belief and health care demands between other cultures and their own made caring more complex, together with their shortage of cultural diversity knowledge, which resulted in further complicating the learning about caring. Participant 1 in focus group 4 stated her experience:

“Once I tried to persuade a patient with chronic renal disease to come to see a doctor and not to turn to minority traditional therapy (such as herbs and alternative therapy) during his discharge education, he seemed ignoring my words and said it was none of my business. I feel frustrated till now, I cannot identify with his beliefs, but how to do as I should and conduct caring, what knowledge should I learn and how to learn, it is a problem”. (group 4)

Furthermore, cultural related problems occurred in clinical practice which were not mentioned in their learning materials or taught by teachers, and were hard to tackle, which often presented a dilemma or left them at a loss. Participant 3 in focus group 4 cited his own experiences:

“When I see patients with ethnic costume and adornment or speaking their own dialects, I am nervous… We are taught that communication and touch are useful ways to care for patients but in this situation there are lots of unknown taboos for me and I am afraid what I do may cause ‘being good and doing harm’. Without preparedness for culture related problems in practice, often I retreat and avoid attending to the patient as much as I can, which will hinder the learning about caring”. (group 4)

## Discussion

This study offers a view of the experience of learning about caring from the perspectives of the baccalaureate nursing students in Yunnan Province. Caring can be learned by observing or experiencing caring incidents because caring incidents can provide positive role models and make us feel the caring atmosphere. This is consistent with findings from previous studies in which care role models are important for learning and also for visible care images, and student nurses learn caring from faculty role models as well as practicing nurses, furthermore, caring begets caring [25-27]. Of particular note, one participant cited her example of experiencing gratitude and acacknowledgement from the patient due to her effort in caring for the patient, which acted as a caring experience, boosted students’ motivation to learn to care. It is in agreement with research findings that when encounters with patients are a rewarding experience that give rise to positive emotions such as a sense of success and self-confidence [28-30], the encounters can increase students’ work motivation [31], hence facilitate their learning about caring while working in the clinical field. Besides, the positive feedback from the patient experienced by the participant showed that they had established a facilitative student-patient relationship focusing on the common good for both the student and the patient, which is seen as supporting the learning of care [32,33]. It is noteworthy that student-patient relationship includes aspects of both caring and learning, which is seen as an important part of meaningful learning and is connected with students’ personal and professional growth [34].

Nurse educators must be fully aware of uncaring incidents and need to be alert to the consequences of such negative experiences [35]. Uncaring experiences are commonly understood as a barrier to professional development of caring. As evidenced by Brainard and Brislen’ study [36] and the research of Byszewski et al. [37], unprofessional conduct by medical educators was the barrier to medical professionalism education. Yet negative role models such as uncaring incidents, inappropriate clinical teachers and unprofessional behaviors in pre-clinical and clinical environment experienced by students were reported by many researchers [37-42]. For medical educators, the question is: how to respond to the negative role models since they continue to exist in today’s health care environment and education system. Ignorance of the problem will lead to devastating consequences. Byszewski et al. [37] pointed out that over time, behaviors that the students previously considered unprofessional, became increasingly more acceptable as students progressed in their training, indicating some erosion of values. Lockwood et al. [43] found that negative role models motivated others by encouraging the avoidance of failure, highlighting prevention strategies, and so were most likely to motivate individuals with prevention goals. They also examined the cross-cultural differences in reactions to positive and negative role models and found that individuals from collectivistic cultures, such as those from China, who had a stronger prevention orientation, would be most motivated by negative role models [44,45]. The findings from our study suggest that students may learn to care from the negative role models by avoiding conducting the same uncaring behaviors they themselves experienced or observed. In this study, uncaring experiences such as coldness and insensitivity [39] provide the chance for students to observe negative role models. Students can observe uncaring behaviors of the negative role models and these leave an imprint in their minds, which generate the most learning about caring and students can learn from the negatives [26,46,47]. But that dose not mean that observation of uncaring behaviors always lead to positive learning. It was also evidenced by Baingana et al. that repeated negative learning experiences may adversely impact the development of professionalism among health professions students [42], which was also mentioned in our study findings. The key is how to transform uncaring observation into constructive learning about caring. In this study, we knew from students’ perspectives that reflective dialogue with competent mentors/supervisors about their negative experiences could transform negative experience into understanding and facilitate the learning about caring, which means that reflective practice can facilitate professional development of caring practice. This is supported by research findings that reflection enhances development of professionalism by offsetting the impact of negative role modeling [48], professionalism being learned by experiences coupled with reflection and discussion [42], and opportunity for reflection must exist hand in hand with role modeling [37]. The core idea underpinning reflective practice is that humans have the capacity to consider in introspective manner, the activities that they are engaged in and then moderate their future activities, which means that reflective practice helps ‘deep’ learning take place [49].

Negative critical incidents may play a constructive role in the choices students make in future circumstances, and experiences with both negative and positive behaviors shape students’ perceptions of the profession and its values [48]. As Hojat reported that student could learn what to do from ‘good ’ docs and learn what not to do from ‘bad’ ones! [50]. The way of learning caring by caring and uncaring experiences is coincident with the core Confucianism in the Chinese culture, which emphasizes being considerate [17]. The experiences of caring and uncaring incidents will help put oneself in the place of another, which can result in ‘what is unpleasant to thyself,do not that to thy neighbor’.

Conducive learning environment was cited as facilitator to the learning about caring. Caring teachers and preceptors act as role modeling, which contribute to the learning about caring. In a nurturing, caring learning environment and positive staff relationship, students can have support from team members, which can facilitate the enhancement of caring [51,52]. Thomas et al. [35] pointed out that the value systems that students are exposed to in practice are important to nurses on a world-wide bases. As evidenced by this study, the caring- centered system, which has resources such as appropriate role models, caring values and ethos as the guidance for the practice, makes it easy to learn how to care for patients. Watson pointed out that the more important power is the power of the group, the community, the learning circle, which suggest the importance of system and environment in the process of learning to care [7].

As evidenced by our findings that lack of directive substantive way of learning reflected students learning needs. A caring attitude is not transmitted from generation to generation by genes [7], instead, caring must be cultivated and taught [6,53]. As participants suggested, only emphasizing the importance of caring in nursing was far from what they needed. A one-sided emphasis of caring as an ideal and moral imperative in nursing can be an unattainable vision, the moral imperative ought to be balanced with students and faculty learning and demonstrating nursing actions and skills that are caring [54]. Developing a caring relationship requires skill and ontological human caring competencies [7]. As nurse educators, we might as well think of the two questions: “Are we teaching our students the art of caring?” and “Should we be teaching caring in a more directive substantive way?” The findings of this study reflected what students really wanted were the teaching of the art of caring and teaching caring in a more directive substantive way. Their desire for the impartation of caring theory, knowledge, skills, attitudes and art is urgent. This is also supported by the findings discussed above that students mostly related their learning from role modeling. In China, caring curriculum in nursing is insufficient and the implementation of strategies to improve caring ability is rare [40]. Lack of directive substantive way of learning indicates that on one hand the formal curriculum needs further development. On the other hand, there is a lacking of teaching and guidance to assist students in fostering critical thinking and reflective practice.

As evidenced by this study, cultural diversity is a challenge in caring and lack of cultural competence is another aspect hampering the learning about caring. In Yunnan, the ethnic minorities are influenced by Chinese culture, western religions, south Asian religions, and some have their ancient religions, which are interwoven together and may differ or even conflict with baccalaureate nursing students’ learned knowledge, hence sometimes undermine the process of learning about caring. The cultural conflicts mentioned by the students such as health care belief, health care demands, linguistic problems, lifestyle, custom and taboo in different cultural encounters made them unable to meet the needs of clients from diverse cultural backgrounds and consequently, they often felt at a loss when learning how to care for people. This should remind the nursing educators and practitioners to develop students’ cultural competence through their teaching [55]. It is also recommended that undergraduate nursing programmes should prepare students to become culturally sensitive [56]. Cultural competence deals with knowledge [57], and in cultural competence model, the greater emphasis is placed on distinguishing characteristics of various ethnic groups [58]. In nursing education, when a multicultural background place like this research setting is shown to us, the mastery of a theoretically finite body of knowledge related to cultural diversity seems overwhelming and impractical. Cultural humility is proposed as a more suitable goal in multicultural medical education [59]. Cultural humility is defined as individuals continually engage in self-reflection and self-critique as lifelong learners and reflective practitioners, which deals with understanding and emphasize on self-awareness, a relationship-centered approach, and learning from patients [58]. By integrating the concept of cultural humility into the curriculum, educators can enhance residents’ ability to provide care that is both culturally sensitive and culturally competent [60]. Furthermore, cultural awareness is important for cultural competence, which involves the recognition of one’s biases, prejudices, and assumptions about individuals who are different [61]. Without being aware of the influence of one’s own culture or professional values, there is risk that the health care provider may engage in cultural imposition practices, which is defined as the tendency of an individual to impose their beliefs, values, and patterns of behavior on another culture [62]. As reflected in our findings, the participant’s negative attitude toward traditional therapy suggests the problem of cultural imposition practice occurs, which has serious ethical and moral implication [62] and will adversely influence cultural awareness. Educators should be alert of the problem of cultural imposition and identify the problem in the early stage to avoid its negative consequence on students’ cultural competence.

## Conclusions

Exploring baccalaureate nursing students’ perspectives on learning about caring shows us some important issues for further thinking. Learning caring by both caring and uncaring experiences warrant our attention. It is without any doubt that caring incidents acting as positive role models can promote the learning about caring, as for uncaring incidents which continue to exist in health care environment and education system, we used to criticize or ignore them, usually we even let students handle them alone, which may leave them with only negative influences and even lead to ethical erosion. The authors recommend that students can learn about caring from uncaring incidents or negative role models in a way of reflective practice.

In seeking students’ views about the facilitators and hampering factors in their learning about caring, we have gained a deeper insight into caring education, particular about the curriculum. To meet the learning needs expressed by the students, formal curriculum concerning caring science, caring knowledge, skills, attitudes and the art of caring should be emphasized in caring education. In addition, the informal and hidden curricula such as role modeling, reflective practice, critical thinking and conducive learning environment play an important role in learning about caring. The findings call for careful choices of teachers, preceptors and medical staff regarding their influence on student nurses and the learning environment are critical.

Furthermore, helping students establish a facilitative nursing student-patient relationship will contribute to their learning about caring. In our setting, cultural diversity presents an additional challenge to learning about caring. Cultural humility involves lifelong learning from patients, which can help students improve their cultural awareness, avoid cultural imposition and prepare students for a lifelong exposure to caring challenges.

### Limitations

One limitation of the study is that purposeful sampling was used and participants were enrolled with the criterion of being talkative and open-minded, so we cannot guarantee that all voices were sufficiently heard. The majority of the participants were Han ethnicity, which may neglect some unique ethnic cultural influences on the learning about caring. Therefore, further exploration in other ethnic minority groups is needed to enrich the findings. Another limitation of the present study is that only students from two colleges in Yunnan Province were included in the study and this may not be representative of all nursing students in China. A relevant follow up study might be to use the same design with students from another country or countries to validate the international nature of the study issue.

### Ethical approval

Kunming Medical College Ethics Review Board.

## Competing interests

The authors declare that they have no competing interests.

## Authors’ contributions

MF contributed to the study conception and design, to data collection, analysis and interpretation, and to the drafting of the paper. JL and JS contributed to the analysis and interpretation of data, and to the drafting of the paper. HL contributed to the study conception and design, and to data analysis, and revised the manuscript. YB contributed to data collection and the drafting of the paper. All authors approved the final manuscript for submission.

## Pre-publication history

The pre-publication history for this paper can be accessed here:

http://www.biomedcentral.com/1472-6920/14/42/prepub
